# Influenza Virus Host Restriction Factors: The ISGs and Non-ISGs

**DOI:** 10.3390/pathogens13020127

**Published:** 2024-01-29

**Authors:** Matloob Husain

**Affiliations:** Department of Microbiology and Immunology, University of Otago, P.O. Box 56, Dunedin 9054, New Zealand; matloob.husain@otago.ac.nz

**Keywords:** influenza, influenza A virus, restriction, antiviral, ISGs, non-ISGs, Mx, IFITMs, TRIMs, cyclophilins, HDACs, ncRNAs

## Abstract

Influenza virus has been one of the most prevalent and researched viruses globally. Consequently, there is ample information available about influenza virus lifecycle and pathogenesis. However, there is plenty yet to be known about the determinants of influenza virus pathogenesis and disease severity. Influenza virus exploits host factors to promote each step of its lifecycle. In turn, the host deploys antiviral or restriction factors that inhibit or restrict the influenza virus lifecycle at each of those steps. Two broad categories of host restriction factors can exist in virus-infected cells: (1) encoded by the interferon-stimulated genes (ISGs) and (2) encoded by the constitutively expressed genes that are not stimulated by interferons (non-ISGs). There are hundreds of ISGs known, and many, e.g., Mx, IFITMs, and TRIMs, have been characterized to restrict influenza virus infection at different stages of its lifecycle by (1) blocking viral entry or progeny release, (2) sequestering or degrading viral components and interfering with viral synthesis and assembly, or (3) bolstering host innate defenses. Also, many non-ISGs, e.g., cyclophilins, ncRNAs, and HDACs, have been identified and characterized to restrict influenza virus infection at different lifecycle stages by similar mechanisms. This review provides an overview of those ISGs and non-ISGs and how the influenza virus escapes the restriction imposed by them and aims to improve our understanding of the host restriction mechanisms of the influenza virus.

## 1. Introduction

The influenza virus is an obligate intracellular pathogen and infects various mammalian and avian species. In humans, the influenza virus causes an acute febrile respiratory disease, influenza, which is commonly known as the ‘flu’. Influenza virus exists in four types: A, B, C, and D. Influenza A virus is the most significant and researched among four types because it infects both mammalian and avian species and causes recurring seasonal epidemics, occasional pandemics, and zoonotic outbreaks. An influenza A virus particle possesses a lipid bilayer envelope, a matrix protein 1 (M1) skeleton underlying the envelope, and a core of eight viral ribonucleoprotein (vRNPs) complexes. The envelope harbors surface antigens, hemagglutinin (HA) and neuraminidase (NA), and an ion channel, matrix protein 2 (M2). The antigenicity of HA and NA glycoproteins continues to evolve due to genetic evolution; hence, influenza A viruses are further subtyped as, e.g., H1N1 and H5N1, based on their HA and/or NA properties. Each vRNP complex is composed of nucleoprotein (NP), three RNA polymerase subunits: polymerase acidic (PA), polymerase basic 1 (PB1), and polymerase basic 2 (PB2), and one of the eight single-stranded, negative-sense RNA genome segments: HA, M, NA, NP, NS, PA, PB1, or PB2 [1].

Influenza virus targets the epithelial cells of the respiratory tract (in mammals) or gastrointestinal tract (in waterfowl) to initiate the infection. For this, influenza virus particle attaches to the host cell by binding the receptor, α-2.6-linked sialic acids (in humans), or α-2.3-linked sialic acids (in avian sp.) through HA. Subsequently, the virus particle is internalized to the host cell, mainly by endocytosis. Through the combined action of low endosomal pH, viral ion channel M2, host proteases, and other factors, the viral envelope fuses with the endosomal membrane, and eight vRNPs are released from the endosomes into the cytoplasm [2,3]. The vRNPs are transported through the cytoplasm and imported into the nucleus, where the viral RNA segment in each vRNP is transcribed and replicated into viral mRNA and viral RNA, respectively. In addition to viral NPs and RNA polymerase subunits (PA, PB1, PB2), various host factors facilitate viral transcription and replication [3]. Then, mature viral mRNAs are exported to the cytoplasm for translation. The viral proteins, HA, M2, and NA, are trafficked to the plasma membrane, whereas the NP, M1, PA, PA-X, PB1, PB2, NS1 (non-structural 1), and nuclear export protein (NEP, formerly known as NS2) are transported to the nucleus. The vRNPs are formed in the nucleus and then exported off the nucleus and trafficked through the cytoplasm to the plasma membrane. The virus assembly occurs at the plasma membrane, and viral progeny is released by budding.

A variety of host factors facilitate and restrict the influenza virus lifecycle at each stage [3]. The host factors that restrict the infection are called host restriction factors or antiviral factors and, broadly, can be of two types: (1) encoded by the interferon-stimulated genes (ISGs) and (2) encoded by the genes that are constitutively expressed or are not stimulated by interferons (non-ISGs). Many host restriction factors in both categories have been identified, some through the latest genetic techniques, such as RNA interference and CRISPR-Cas9 (Supplementary Table S1), and characterized to restrict influenza virus infection. This review compiles those host restriction factors and summarizes their infection restriction mechanisms. Also, this review identifies any strategies the influenza virus employs to escape the restriction imposed by host restriction factors.

## 2. ISGs

The expression of ISGs, as the name suggests, is induced by interferons. Interferons are the first line of defense molecules produced by host cells after sensing the virus infection through pattern recognition receptors. The existence of ISGs was first detected in the later part of the 20th century [4,5]. Since then, several hundreds of ISGs have been identified [6] and characterized to inhibit the infection of many viruses [7]. Likewise, many ISGs, encoding both proteins and non-coding RNAs (ncRNAs), have been identified to express in response to the influenza virus infection and restrict its infection at different stages of the viral lifecycle. Table 1 summarizes the individual ISGs known to restrict influenza virus infection at different stages of the lifecycle with their antiviral targets.

### 2.1. Mx Proteins

The Mx (myxovirus) gene encoding an ~75 kDa protein was the first ISG to be discovered to confer resistance to influenza virus infection [8,9,10,11,12]. Except for chickens [13,14,15,16], Mx proteins in the majority of influenza virus hosts, e.g., humans [17], pigs [18,19], and horses [20], exhibit antiviral activity. Mx proteins are dynamin-like GTPases [21,22,23], which oligomerize into ring-like structures [24,25,26,27,28] and target influenza virus vRNPs to exert their antiviral function [29,30]. Human Mx protein interacts with viral NP and PB2 to sense and sequester the incoming vRNPs in the cytoplasm and inhibit their nuclear import and subsequent viral RNA transcription and replication [20,29,30,31,32,33]. Human Mx protein is a barrier to the zoonotic transmission of avian influenza viruses and recently discovered bat influenza viruses to humans [34,35,36,37,38,39]. To escape this barrier, avian influenza viruses acquire human-adaptive mutations in their NPs or increase their RNA polymerase activity or vRNP nuclear export [35,38,39,40,41,42,43]. Some influenza viruses can also escape this barrier in humans and animals carrying naturally occurring Mx allele variants, which either lack or exhibit reduced antiviral activity [18,44,45,46,47,48].

### 2.2. IFITM Proteins

The IFITM (interferon-inducible transmembrane) genes encoding 14–16 kDa proteins were identified as ISGs around the same time as the Mx gene [49,50,51,52]. However, the antiviral function of IFITM proteins 1, 2, and 3 during influenza virus infection was discovered much later in a genomic screen [53]. IFITM proteins are broad host restriction factors of the influenza virus, as IFITMs from multiple host tissues and species (including bacteria [54]) are capable of inhibiting influenza virus infection [55,56,57,58,59,60,61,62,63,64,65,66,67,68,69]. IFITMs 1, 2, and 3 are closely related proteins and share 70–90% homology, and all three inhibit influenza virus infection by inhibiting its entry to the host cells [53]. IFITM3 is a type II transmembrane protein and localizes to the endosomes and lysosomes, where it interacts with influenza virus HA and prevents the fusion of viral envelope with the endosomal membrane by interfering with lipid homeostasis, consequently preventing vRNP release into the cytoplasm [70,71,72,73,74,75,76,77,78,79,80,81].

The antiviral activity of IFITM3 is regulated by posttranslational modifications like palmitoylation, ubiquitination, and methylation [61,82,83,84,85,86,87,88]. Specifically, the palmitoylation of IFITM3 promotes its antiviral activity by enhancing its membrane affinity and endosomal localization [61,82,83,84,89]. In contrast, the ubiquitination of IFITM3 reduces its antiviral activity by decreasing its stability and localization to the endosomes [83,87]. Also, the methylation of IFITM3 reduces its antiviral activity and influenza disease severity [85,88]. The phosphorylation of IFITM3 reduces its ubiquitination and may indirectly promote its antiviral activity [86]. These findings indicate that the influenza virus potentially employs the ubiquitin ligases, e.g., NEDD4 [87], and methyltransferases, e.g., SET7 [88], to antagonize the antiviral function of IFITM3 and escape IFITM3 restriction. Furthermore, avian influenza A virus subtypes H5N1 and H7N9 may escape IFITM3 restriction in cells with inefficient endosomal acidification [90].

Influenza virus may also escape IFITM3 restriction and cause severe disease in humans carrying single nucleotide polymorphisms (SNPs) in the IFITM3 gene [56,91,92]. The IFITM3 allele carrying SNP rs12252-C encodes an N-terminally truncated IFITM3 variant, which is incapable of localizing to the endosomes and allows the influenza virus to escape IFITM3 restriction [56,73,93,94]. Consequently, rs12252-C has been associated with severe influenza disease [56]. However, the evidence of this association has been found in studies involving the cohorts mainly from Asian ethnicity [92,95,96,97,98,99,100,101,102] and not from other ethnicities [103,104,105,106,107,108,109]. Further, the SNPs in IFITM1 are not associated with influenza disease severity [110].

### 2.3. TRIM Proteins

TRIM (tripartite motif) proteins are a large family of proteins that comprise a conserved architecture known as RBCC (a RING finger domain, one to two B-box domains, a coiled coil domain, and a variable C-terminus) [111,112]. Among TRIMs, TRIM19, also known as promyelocytic leukemia (PML) protein, was the first to be identified as an ISG [113]. Soon after, it was discovered to inhibit influenza virus infection [114]. Now, over 80 TRIMs are known [112], of which at least 27 TRIMs have been identified as ISGs [115]. In addition to TRIM19, TRIMs 14, 21, 22, 25, 35, and 56 have been shown to inhibit influenza virus infection [116,117,118,119,120,121,122,123]. TRIMs are E3 ubiquitin ligases and are part of the ubiquitin–proteasome system, which degrades proteins. Hence, most TRIMs exert their antiviral function by targeting the viral proteins for degradation. Specifically, TRIM14 [119] and TRIM22 [116] target the NP, TRIM21 targets the M1 [124], and TRIM35 targets the PB2 [120] for ubiquitin ligase-dependent degradation. However, the NP of some influenza A virus H1N1 subtypes is resistant to TRIM22-mediated restriction [125]. TRIM25 [118,122] and TRIM56 [117] interfere with viral RNA synthesis or stability though in an E3 ligase-independent manner. Also, TRIM25 has been reported to inhibit influenza virus infection by facilitating its RIG-I-mediated host sensing in a ubiquitin ligase-dependent manner [126,127,128,129]. However, influenza virus antagonizes the latter function of TRIM25 via NS1 protein, which is the main influenza virus virulence factor that antagonizes host defenses. NS1 binds TRIM25 and interferes with its ubiquitin ligase activity [126,127,128,129,130].

### 2.4. OAS Proteins

OAS (2′,5′-oligoadenylate synthetase) proteins 1, 2, and 3, and OAS-like (OASL) protein were among the first ISGs to be discovered [131,132]. OAS 1, 2, and 3 are activated by sensing the viral RNA and then convert the ATP to 2′,5′-oligoadenylate [132], which, in turn, activates the ribonuclease (RNase) L [133]. Subsequently, RNase L restricts influenza virus infection by degrading the viral RNA [134,135,136]. However, OASL restricts influenza virus infection in an RNase L-independent manner [137]. In turn, influenza virus escapes the OAS-mediated restriction via NS1, which competes with OAS proteins for viral RNA binding [134]. Furthermore, the influenza virus may escape this restriction in humans carrying the SNP rs10774671 in OAS1 gene [138].

### 2.5. IFIT Proteins

The IFIT (interferon-induced proteins with tetratricopeptide repeats) family has four proteins, IFITs 1, 2, 3, and 5 (or ISGs 56, 54, 60, and 58, respectively), which have been characterized in humans [139]. IFIT1 is the prototypic member of the family and was the first to be identified as an ISG in the IFIT family [140,141], followed by the rest [139]. The indication of an antiviral function of human IFITs 1, 2, and 3 during influenza virus infection was first discovered in a proteomic screen [142]. Later, it was demonstrated that human IFITs 1, 2, and 3 and avian IFIT5 exhibit antiviral properties during influenza virus infection [143,144,145,146,147]. The human and chicken IFITs exert their antiviral function by sequestering the viral RNA by binding its 5′-triphosphate group, called PPP-RNA [142,145,148], whereas the duck IFIT sequesters viral NPs [144]. However, Pinto et al. found no antiviral activity of human IFIT1 during influenza virus function [149], while Tran et al. found influenza virus rather exploiting the RNA binding property of IFIT2 to promote viral mRNA translation [150].

### 2.6. hGBP Proteins

hGBPs (human guanylate-binding proteins), like Mx proteins, belong to the GTPase family [151,152], and hGBPs -1, -2, -3, and -5 have been shown to inhibit influenza virus infection [153,154,155,156]. hGBP-3 exerts its antiviral function by targeting the viral RNA polymerase activity [153], whereas hGBP-2 and hGBP-5 target the host furin protease, which primes the HA of the highly pathogenic influenza A viruses, like H5N1 subtype, for infection [155]. Nevertheless, influenza virus NS1 antagonizes hGBP-1 by inhibiting its GTPase activity [156].

### 2.7. Tetherin

Tetherin, also known as BST-2/CD317/HN1.24, is a GPI-anchored transmembrane protein [157] and restricts virus infection by tethering the viral progeny to the cell surface. The antiviral role of tetherin during influenza virus infection is inconclusive and has been controversial. However, tetherin expression is induced in influenza virus-infected cells in an interferon-dependent manner [158]. Human tetherin was observed to effectively tether the budding influenza virus-like particles to the plasma membrane [159,160,161,162]; however, the same was not observed with live influenza virus particles [158,159,163] or tetherin from other host species [164,165]. In other studies, tetherin was observed to inhibit the influenza virus release [161,162,166,167], but this restriction was either NA-dependent [161,167] or countered by M2 protein, which facilitated the downregulation of tetherin on the cell surface [162].

### 2.8. ISG15

The ISG15 gene [168] encodes a 15-kDa protein [169], which inhibits influenza virus infection [170] by targeting critical viral [171,172] and host [173] proteins. ISG15 is a ubiquitin-like protein [169] and is conjugated to target proteins by sequential action of several conjugation enzymes, some of which are also ISGs [174,175,176,177,178,179]. This process is also called ‘ISGylation’. ISG15 ISGylates influenza virus NS1 protein and cripples its ability to perform various antagonistic functions [171,172]. Further, the ISGylation of host protein Tsg101 inhibits the trafficking of viral HA to the plasma membrane, the site of influenza virus assembly [173].

### 2.9. PKR

PKR (protein kinase R) is a dsRNA-activated serine/threonine protein kinase and phosphorylates the eukaryotic translation initiation factor 2 (eIF-2α); this leads to the inhibition of the initiation of global protein synthesis [180]. This leads to the inhibition of viral protein synthesis too, and consequently, the influenza virus infection [181,182]. Influenza virus counteracts this restriction through NS1, which binds to dsRNA and blocks PKR activation [182,183,184]. Influenza virus NP also can block PKR activation by activating the cellular PKR inhibitor, P58 [185]. 

### 2.10. Other Proteins

CEACAM1 (carcinoembryonic antigen-related cell adhesion molecule 1) expression was first shown to be induced by interferon-gamma [186]. CEACAM1 inhibits influenza virus infection by suppressing the mTOR (mammalian target of rapamycin) activity, consequently inhibiting the global protein synthesis in infected cells [187].

IFI16 (interferon γ-inducible 16) is a ~80-kDa nucleic acid-binding protein [188,189]. It is a PYHIN (pyrin and hematopoietic interferon-inducible nuclear (HIN) domain) family protein and was initially identified as an intracellular DNA sensor [190]. Recently, IFI16 has been discovered to inhibit influenza virus infection by sensing the viral RNA and promoting the RIG-I-mediated innate antiviral response [191,192].

ISG20 (interferon-stimulated gene 20), as the name suggests, is a 20-kDa protein with 3′ to 5′ exonuclease activity that is specific for single-stranded RNA [193,194]. ISG20 inhibits influenza virus infection by interfering with viral RNA transcription and replication [195,196]. 

MOV10 (Moloney leukemia virus 10) is a member of the RNA helicase superfamily [197], and its expression can be stimulated by interferons [7]. MOV10 inhibits influenza virus infection by binding to NP and sequestering the incoming vRNPs in the cytoplasm, consequently inhibiting their nuclear import [198,199,200]. However, the antiviral function of MOV10 is independent of its RNA helicase activity [199,200].

MUC1 (mucin 1) is a member of mucins, a family of highly glycosylated proteins that are expressed on the surface of respiratory epithelial cells, which are the target of influenza virus infection. MUC1 potentially acts as a receptor decoy and inhibits influenza virus infection by binding to virus particles and blocking their attachment to target cells [201,202,203].

NCOA7 (nuclear receptor coactivator 7) expression is induced by the interferon-beta [204]. NCOA7 inhibits influenza virus infection by inhibiting the fusion of the viral envelope with the endosomal membrane during entry [205].

p21 is a cyclin-dependent kinase inhibitor and inhibits influenza virus infection by interfering with viral RNA polymerase activity [206].

Serpin 1 or plasminogen activator inhibitor 1 (PAI-1) inhibits influenza virus infection by neutralizing host proteases, like trypsin, and preventing the cleavage of HA, which is required for influenza virus entry [207]. However, influenza virus may escape this restriction in humans carrying the naturally occurring SNP rs6092 in serpin 1 gene [207].

SERTAD3 (SERTA domain containing 3), also called RBT1 (replication protein A binding transactivator 1), is one of the SERTA family transcription factors, and its expression is induced by interferons [208]. SERTAD3 inhibits influenza virus infection by disrupting the formation of the viral RNA polymerase complex [208].

SLFN11 and SLFN14 are Schlafen family proteins and possess an RNA helicase domain [209]. SLFN11 and SLFN14 expression is induced by interferons, and both inhibit influenza virus infection by contributing to host innate defenses [210,211].

SPOCK2 (SPARC/osteonectin CWCV and Kazal-like domains 2) or testican 2 is a secreted proteoglycan, and it inhibits influenza virus infection by blocking the attachment of virus particles to the cell surface [212].

RABGAP1L (RAB GTPase-activating protein 1-like) or TBC1D18 (Tre2/Bub2/Cdc16 (TBC)-domain-containing 18) protein restricts influenza virus infection by disrupting the endosome function hence virus entry [213].

Viperin (virus inhibitory protein, endoplasmic reticulum-associated, interferon-inducible) protein [214], also called RSAD2, inhibits influenza virus infection by disrupting the lipid rafts on the plasma membrane and inhibiting the viral progeny release [215,216].

ZAP (zinc finger antiviral) or ZC3HAV1 (Zinc finger CCCH-type antiviral 1) protein exists in two forms, short (ZAPS) and long (ZAPL), and both forms exhibit anti-influenza virus properties [217,218,219]. The ZAPS exerts its antiviral function by promoting the degradation of viral mRNA but is antagonized by the NS1, which competes with ZAPS for viral mRNA binding [218]. Whereas ZAPL promotes the degradation of viral PA and PB2 and is antagonized by viral PB1, which binds ZAPL and displaces PA and PB2 [217].

### 2.11. ncRNAs

Much of the human genome is transcribed into non-coding RNAs (ncRNAs), which do not translate into a protein. Based on their length, these ncRNAs are called microRNAs or miRNAs (~22 nucleotides), small-interfering RNAs or siRNAs (21–25 nucleotides), piwi-related RNAs or piRNAs (24–33 nucleotides), vault RNAs or vtRNAs (80–150 nucleotides), or long non-coding RNAs or lncRNAs (>200 nucleotides). Further, some lncRNAs exist as covalently closed circular RNAs or circRNAs. Many ncRNAs are upregulated in response to influenza virus infection and inhibit infection by targeting the viral proteins and critical host proteins [220].

lncRNAs are the prominent form of ncRNAs that have been identified to be upregulated in response to influenza virus infection or interferon treatment [221,222,223,224,225,226,227,228]. These lncRNAs inhibit influenza virus infection primarily by strengthening the antiviral state in infected cells through various mechanisms, e.g., stabilization of the RIG-I–TRIM25 complex for host sensing of the influenza virus [222], epigenetic modifications of the regulatory regions of innate response genes [225,227], and manipulation of the regulators (including miRNAs) of interferon signaling [221,223,226,228].

Also, circRNAs, circVAMP3, and AIVRs are upregulated in response to influenza virus infection and restrict the infection by different mechanisms [229,230]. The circVAMP3 acts as a decoy to viral NP and NS1 and interferes with their function [230], while the AIVR sequesters a microRNA, which degrades an enhancer of the interferon production [229].

In addition, the miRNAs, miR-101, miR-485, ssc-miR-221-3p, and ssc-miR-222, have been identified to be upregulated in response to influenza virus infection and inhibit infection by distinct mechanisms [231,232,233]. The miR-101, like ISG CEACAM1 [187], inhibits influenza virus infection by targeting the mTOR pathway [232], whereas miR-485 targets host RIG-I and viral PB1 and reduces their mRNA levels [231]. Further, swine ssc-miR-221-3p and ssc-miR-222 may restrict the interspecies transmission of avian influenza viruses to pigs by targeting their viral RNA [233].

## 3. Non-ISGs

Host proteins encoded by the constitutively expressed genes (called non-ISGs) also restrict influenza virus infection. The majority of these non-ISGs have been identified either through co-immunoprecipitation followed by mass spectrometry analyses or yeast-two hybrid, RNA interference, and CRISPR-Cas genetic screens. The non-ISGs known to restrict influenza virus infection at different stages of the lifecycle and their antiviral targets are summarized in Table 2.

### 3.1. Cyclophilins

Cyclophilins are ubiquitously present peptidyl-prolyl cis-trans isomerases, which chaperon the folding of proteins [234]. Multiple cyclophilins are known, and cyclophilins A, D, and E have been discovered to inhibit influenza virus infection [235,236,237,238,239]. Cyclophilin A exerts its antiviral function by targeting and degrading the M1 protein [235,236,237,240]. Also, cyclophilin A promotes the RIG-I-mediated sensing of the influenza virus [241]. Cyclophilin E interacts with NP and interferes with the formation of the vRNP complex [242], whereas cyclophilin D helps increase influenza disease tolerance [239].

### 3.2. TRIM Proteins

Some TRIMs, e.g., TRIM16, 32, and 41, are also non-ISGs, but, like the ISG TRIMs, they inhibit influenza virus infection in a ubiquitin ligase-dependent manner [243,244,245]. TRIM32 and TRIM41 restrict influenza virus infection by ubiquitinating and degrading the PB1 [243] and NP [244], respectively, whereas TRIM16 ubiquitinates nuclear factor erythroid 2-related factor 2 (NRF2) and reduces the oxidative stress in infected cells [245].

### 3.3. DEAD-Box RNA Helicases

DDX3, DDX21, and DDX30 belong to the DEAD-box RNA helicase family and inhibit influenza virus infection by three different mechanisms [246,247,248,249]. DDX3 promotes stress granule formation and activates the NLRP3 inflammasome [247,249]. DDX21 binds PB1 and interferes with the RNA polymerase activity, whereas DDX30 binds NS1. In turn, influenza virus counteracts the DDX3- and DDX21-mediated restriction via NS1, which inhibits the stress granule formation [247,249], binds to DDX21, and displaces PB1 [248].

### 3.4. MARCH Proteins

MARCH (membrane-associated RING-CH-type) proteins are RING (really interesting novel gene) finger E3 ligases and are known to downregulate the expression of cellular proteins on the cell surface. MARCH1 and MARCH8 inhibit influenza virus infection though it is unclear if, like in case of other enveloped viruses, they downregulate the expression of influenza virus membrane proteins on the cell surface [250,251,252]. However, MARCH8 has been identified to block the furin-mediated cleavage of HA of avian influenza A virus H5N1 subtype [250].

### 3.5. Translation Factors

The eukaryotic translation initiation factor 4B (eIF4B) inhibits influenza virus infection indirectly by promoting the translation of ISGs, like ISG15 and IFITM3 [253]. But, influenza virus overcomes this restriction by promoting the lysosome-mediated degradation of eIF4B [253]. The eukaryotic translation elongation factor 1 delta (eEF1D) inhibits influenza virus infection by a different mechanism; it impedes the nuclear import of vRNPs by impairing the interaction of the NP and PB1 with their nuclear receptors [254].

### 3.6. HDACs

HDACs (histone deacetylases), also known as lysine deacetylases (KDACs), are the erasers of acetylation from proteins and have been discovered to inhibit influenza virus infection. So far, eighteen mammalian HDACs are known and divided into four classes. HDACs 1, 2, 3, and 8 belong to class I, HDACs 4, 5, 6, 7, 9, and 10 belong to class II, and HDAC11 belongs to class IV. Class III HDACs contain seven members, which are called sirtuins (SIRT 1–7). Multiple HDACs from each class inhibit influenza virus infection by various mechanisms [255,256,257,258,259,260,261,262,263,264]. Most HDACs exert their antiviral function by promoting the expression of interferons and ISGs, like IFITM3, ISG15, ISG20, and Viperin, by deacetylating various innate immune factors and effectors [255,256,257,260,262,265,266,267]. In addition, HDAC6 deacetylates viral PA and promotes its degradation [268]. Further, SIRT2 deacetylates G6PD (glucose-6-phosphate dehydrogenase), which reduces the oxidative stress in infected cells; notably, oxidative stress is beneficial for the influenza virus growth [269].

Influenza virus overcomes the antiviral function of HDACs by downregulating their expression at both mRNA and protein levels [255,256,257,260,261,269,270], as well as their enzymatic activity [255,258,271]. Specifically, influenza virus downregulates the expression of HDAC4 and HDAC6 mRNA via PA [260,270] and HDAC8 mRNA via miR-21-3p [261]. Further, influenza virus exploits the host proteasome and caspases to promote the degradation of HDAC1 and HDAC2 [255,256] and HDAC4 and HDAC6 [260,270], respectively.

### 3.7. Other Proteins

Annexin 6, a calcium-dependent membrane-binding protein involved in membrane organization, inhibits influenza virus infection by interacting with M2 and interfering with the budding of viral progeny [272,273].

APOE (apolipoprotein E) restricts influenza virus infection by interfering with membrane cholesterol homeostasis and inhibiting the virion attachment to the cell surface [274].

B3GAT1 (beta-1,3-glucuronyltransferase 1) and B4GALNT2 (beta-1,4-N-acetyl-galactosaminyltransferase 2) restrict influenza virus infection by targeting the sialic acid receptor. B3GAT1 reduces the expression of sialic acid [275], whereas B4GALNT2 modifies the sialic acid [276,277,278]. Both events preclude the attachment of influenza virus particles to the cell surface.

BTN3A3 (butyrophilin subfamily 3 member A3) inhibits the infection of, specifically, avian influenza viruses by interfering with the replication of viral RNA [279]. Hence, BTN3A3 is a barrier to the transmission of avian influenza viruses to humans. However, like Mx proteins, zoonotic avian influenza viruses escape this barrier by acquiring escape mutations in their NPs [279].

β-TrCP (β-transducin repeat-containing protein), an E3 ligase, inhibits influenza virus infection, but viral NS1 overcomes the β-TrCP restriction by inducing its degradation in infected cells [280].

Cyclin D3, a key cell cycle regulator, like annexin 6, interacts with M2 and inhibits its interaction with M1, consequently inhibiting the formation of influenza virus progeny [281].

Galectin-1 is an S-type lectin and is secreted extracellularly in the lungs. It binds influenza virus particles and inhibits their attachment to the cell surface [282], consequently inhibiting the infection and reducing disease severity [282,283,284]. Further, galectin-1 gene variants, SNPs rs4820294 and rs13057866, express galectin-1 at a higher level and protect the humans carrying those SNPs from severe influenza virus infection [283].

HAX-1 (HCLS1-associated X1), an anti-apoptotic protein, inhibits influenza virus infection by binding to PA and blocking its nuclear import [285]. However, mostly zoonotic avian influenza viruses are sensitive to the HAX-1-mediated restriction [286]. Nevertheless, zoonotic avian influenza viruses can overcome this restriction via viral PB1-F2, which also binds to HAX-1 and competes with PA for this binding [286,287].

hnRNPAB (heterogeneous nuclear ribonucleoprotein A/B) restricts influenza virus infection by promoting the nuclear retention of viral mRNA [288,289].

Hsp70 (heat shock protein 70) has been described to inhibit influenza virus infection by interacting with PB1 and PB2 and interfering with vRNP integrity, which results in the interference of viral RNA replication [290]. But, another study claimed that HsP70-PB1-PB2 interaction promotes viral RNA replication [291].

JADE3 (Jade family PHD zinc finger 3), also called PHF16 (PHD zinc finger 16), restricts influenza virus infection and by activating the NF-kB signaling [292].

MMP3 (matrix metalloproteinase 3) restricts influenza virus infection by translocating to the nucleus and promoting the expression of antiviral cytokines and chemokines [293].

NF90 (nuclear factor 90) has been described to inhibit influenza virus infection by three mechanisms: (1) interacting with the NP and interfering with viral RNA transcription and replication [294], (2) negatively regulating the phosphorylation of ISG PKR [295], and (3) antagonizing the NS1 [296].

PGRMC1 (progesterone receptor membrane component 1) restricts influenza virus infection by antagonizing the ubiquitination-mediated activation of RIG-I. Potentially, it determines the neurotropism of influenza viruses too [297].

PKP2 (plakophilin 2) protein, mainly known for the formation of desmosomes and stabilization of cell junctions, binds to PB1. It restricts influenza virus infection by competing with PB2 for binding to PB1 and impeding RNA polymerase activity [298].

PIAS1 (protein inhibitor of activated STAT1), a SUMO E3 ligase, inhibits influenza virus infection by SUMOylation-mediated degradation of PB2 [299].

Pirh2 (p53-induced RING-H2), an E3 ubiquitin ligase, inhibits the infection of human but not avian influenza viruses. Pirh2 ubiquitinates the NP, which interrupts the NP-PB2 interaction and, consequently, the formation of a vRNP complex [300].

PSMB4 (proteasome subunit beta type 4) restricts influenza virus infection by targeting NS1 and facilitating its degradation in infected cells [301].

RTF2 (replication termination factor 2) is a nucleus-localized protein and restricts influenza virus infection by inhibiting the viral transcription and promoting the interferon response [302].

RSK2 (ribosomal protein S6 kinase alpha 2), a mitogen-activated protein kinase, inhibits influenza virus infection by promoting the innate antiviral response [303].

SERINC5, one of the five SERINC (serine incorporator) family membrane proteins, inhibits influenza virus infection by interfering with the fusion of the viral envelope and endosomal membrane during entry [304,305].

TBC1D5 (TBC1 domain family member 5), an autophagy regulator, restricts influenza virus infection by promoting the lysosome-mediated degradation of M2 [306].

TET2 (ten-eleven translocation 2), a methylcytosine dioxygenase, inhibits influenza virus infection by enhancing the expression of STAT1 and consequently the expression of various ISGs via DNA demethylation [307]. However, influenza virus counters the TET2-mediated restriction by downregulating the TET2 expression through its host shutoff protein PA-X [307].

TRA2A (transformer 2 alpha homolog), an mRNA splicing regulator, restricts the infection of avian influenza viruses but not the human influenza viruses in humans. Human TRA2A binds to the ISS (intronic splicing silencer) motif of avian influenza virus M mRNA and inhibits its splicing into M1 mRNA and M2 mRNA [308]. Some avian influenza viruses may have escaped the TRA2A restriction and adapted to humans by mutating the ISS motif in their M genes [308].

TUFM (Tu elongation factor, mitochondrial) protein acts as a barrier to interspecies transmission of avian influenza viruses to humans. TUMF binds to avian influenza virus PB2 in mitochondria and induces autophagy, which, in turn, restricts avian influenza virus growth in human cells [309]. However, the E627K mutation in PB2 of avian influenza viruses impedes the binding of TUMF to PB2 and allows avian influenza viruses to escape this restriction and multiply in human cells [309].

ZMPSTE24 (zinc metallopeptidase STE24) is an effector of IFITMs and inhibits influenza virus infection by facilitating the antiviral function of IFITMs in endosomes [310,311]. However, the antiviral function of ZMPSTE24 is independent of its protease activity [310,311].

### 3.8. ncRNAs

In the non-ISG category, mostly miRNAs have been identified to inhibit influenza virus infection. miR-323, miR-491, miR-654 [312], and miR-324-5p [313] inhibit influenza virus infection by targeting and degrading the PB1 RNA and miRNA let-7c [314] by targeting and degrading the M RNA. Whereas hsa-mir-127-3p, hsa-mir-486-5p, hsa-mir-593-5p, and mmu-mir-487b-5p target multiple viral RNAs [315].

miR-206 [316], miRNA-30 [317], and miR-221 [318] inhibit influenza virus infection by promoting the antiviral state in host cells by various mechanisms. miRNA-30 [317] and miR-221 [318] suppress the expression of SOCS1 and SOCS3 genes, which restrict type I interferon signaling, while miR-206 suppresses the expression of tankyrase, a poly (ADP-ribose) polymerase [316]. miR-29a inhibits influenza virus infection by targeting the frizzled 5 protein in the Wnt signaling pathway [319].

## 4. Summary

A plethora of host restriction factors, ISGs and non-ISGs, have been identified, which restrict influenza virus infection by inhibiting the viral attachment, entry, synthesis, assembly, and release, and strengthening the host innate antiviral response (Table 1 and Table 2). However, the influenza virus seems to have the upper hand and effectively antagonizes the restrictions imposed by these factors. Basically, there are three strategies that influenza virus employs to perform this: (1) the acquisition of escape mutations in viral proteins, like NP, targeted by the restriction factors, (2) the downregulation of the expression of restriction factors at both mRNA and protein levels via viral endonucleases, PA and PA-X, or host factors, ncRNAs, proteasome, lysosome, and caspases, and (3) the sequestration or interference of the restriction factors by viral proteins, like NS1 (Table 3). Furthermore, the genetic diversity of some restriction factors (galectin-1, IFITM3, Mx, OAS-1, Serpin-1) in various hosts and human populations also helps the influenza virus to escape the host restriction. Nevertheless, an exhaustive list of the influenza virus host restriction factors with their restriction mechanisms is yet to be compiled. A comprehensive knowledge of host restriction factors and influenza virus interplay is critical for designing targeted antiviral interventions to overcome the existing and newly emerging influenza virus variants.

## Figures and Tables

**Table 1:** ISGs restricting the influenza virus lifecycle at different stages.

Viral Lifecycle Stage	ISGs (Antiviral Targets)
Attachment	MUC1, SPOCK2 (virions);
Entry	hGBP-2, hGBP-5, Serpin 1 (host protease); IFITM 1, 2, and 3, NCOA7, RABGAP1L (viral–endosomal membrane fusion)
Synthesis	CEACAM1, miRNA101 (mTOR); hGBP-3, p21, SERTAD3 (viral RNA polymerase); IFIT 1, 2, and 3, ISG20, OAS 1, 2, 3, and L, TRIM 25 and 56 (viral RNA); MOV10, Mx (vRNP); PKR (eIF-2α); ZAPS (viral mRNA)
Assembly	ISG15 (Tsg101); TRIM 14 and 22 (NP); TRIM21 (M1); TRIM35 (PB2); ZAPL (PA, PB2)
Release	Tetherin (virions); Viperin (lipid rafts)
Innate response	IFI16, miRNA485 (RIG-I); circVAMP3, ISG15 (NS1); lncRNAs (RIG-I, interferon); SLFN 11 and 14

**Table 2:** Non-ISGs restricting the influenza virus lifecycle at different stages.

Viral Lifecycle Stage	Non-ISGs (Antiviral Targets)
Attachment	APOE (cell membrane); B3GAT1, B4GALNT2 (sialic acid); galectin-1 (virions)
Entry	MARCH8 (host protease); SERINC5, ZMPSTE24 (viral–endosomal membrane fusion)
Synthesis	BTN3A3, RTF2, hsa-mir-127-3p, -486-5p, -593-5p, and -487b-5p (viral RNA); eEF1D (vRNP); HAX-1 (PA); hnRNPAB (viral mRNA); DDX21, Hsp70, PKP2, TUFM (PB1, PB2); NF90 (NP); microRNA let-7c, TRA2A (M mRNA); miR-323, -491, -654, and -324-5p (PB1 RNA)
Assembly	Cyclophilin E (NP); cyclophilin A (M1); PIAS1 (PB2); Pirh2 (NP); TRIM16 (NRF2); TRIM 32, 41 (PB1); TBC1D5 (M2); β-TrCP
Release	Annexin 6, cyclin D3 (M2)
Innate response	Cyclophilin A, PGRMC1 (RIG-I); DDX30, PSMB4 (NS1); eIF4B, HDACs (interferons, ISGs); JADE3; MMP3; NF90 (PKR, NS1); RSK2; TET2 (STAT1); miR-29a (frizzled 5); miR-30 and -221 (SOCS 1 and 3); miR-206 (tankyrase)

**Table 3:** Influenza virus strategies to escape the restriction from ISGs and non-ISGs.

Strategies	ISGs	Non-ISGs
Mutations in viral proteins	Mx, TRIM22	BTN3A3, TRA2A, TUFM
Downregulation of expression	IFITM3, Tetherin	β-TrCP, HDACs, TET2
Sequestration or interference	hGBP-1, OAS, PKR, TRIM25, ZAP	DDX3, DDX21, HAX-1

## Data Availability

Not applicable.

## Supplementary Materials

The following supporting information can be downloaded at https://www.mdpi.com/article/10.3390/pathogens13020127/s1, Table S1. Influenza virus restriction factors identified by RNA interference or CRISPR-Cas9 screenings/techniques.
