# Supplementary material for: Influenza Virus Host Restriction Factors: The ISGs and Non-ISGs
Source: Pathogens. 2024 Jan 29;13(2):127. doi: 10.3390/pathogens13020127 (PMC10893265; doi:10.3390/pathogens13020127)
Supplement: Supplementary file 1 [file pathogens-13-00127-s001.zip › pathogens-2806690-supplementary.pdf]

Table S1. Influenza virus restriction factors identified by RNA interference or CRISPR-Cas9 screenings/techniques.

Screenings/Techniques	ISGs	Non-ISGs
RNA interference	IFITM 1, 2, & 3, p21, RABGAP1L	HDACs
CRISPR-Cas9	MUC1, OAS3	Annexin 6, APOE, B3GAT1, B4GALNT2, JADE3, miR-29a, PIAS1, RTF2
